# Spillover effects of pro-environmental behavior in the Metaverse

**DOI:** 10.3389/fpsyg.2026.1763015

**Published:** 2026-02-18

**Authors:** Xiaoming Wang, Keqin Zhang, Jie Yu

**Affiliations:** 1 Cognition and Human Behavior Key Laboratory of Hunan Province, School of Education Science, Hunan Normal University, Changsha, China; 2 Office of Infrastructure Construction, Hunan University of Technology and Business, Changsha, China; 3 Mental Health Education and Counseling Center, Hunan Normal University, Changsha, China

**Keywords:** environmental self-identity, guilt, labeling, Metaverse, pro-environmental behavior, spillover effects

## Abstract

Existing research has extensively explored the spillover effects of pro-environmental behavior. However, systematic research regarding the spillover effects and psychological mechanisms of pro-environmental behavior in the Metaverse remains scarce. This study investigates the spillover effects of pro-environmental behavior in the Metaverse on real-world pro-environmental behavior and the underlying mediating mechanisms. Additionally, it explores the intervention effect of “environmentalist” labeling. Study 1 examined the spillover effects of Metaverse pro-environmental behavior on real-world pro-environmental behavior and identified the associated mediating mechanisms. The results revealed that: (1) participants engaging in pro-environmental behavior in the Metaverse exhibited stronger environmental self-identity, which subsequently promoted real-world pro-environmental behavior, thereby generating a positive spillover effect; (2) simultaneously, these participants reported lower levels of guilt, which led to a reduction in subsequent real-world pro-environmental behavior, indicating a negative spillover effect; and (3) as both positive spillover and negative spillover pathways were activated concurrently, pro-environmental behavior in the Metaverse did not yield a significant total spillover effect on real-world pro-environmental behavior. Study 2 further investigated the intervention effect of “environmentalist” labeling. The results indicated that assigning the “environmentalist” label to participants performing pro-environmental behavior in the Metaverse enhanced their environmental self-identity without significantly reducing guilt. Consequently, this intervention significantly enhanced the total spillover effect. These findings suggest that spillover effects can extend from the Metaverse to the real world, with environmental self-identity and guilt mediating the positive spillover and negative spillover pathways, respectively. Furthermore, “environmentalist” labeling shifts the total spillover effect from non-significant to positive by reinforcing the positive spillover mediated by environmental self-identity and mitigating the negative spillover mediated by guilt.

## 1 Introduction

In recent years, environmental degradation and global disasters have intensified public concern over pollution and environmental sustainability. Addressing environmental challenges necessitates a deep understanding of human behavior, as many environmental issues are intrinsically linked to the actions, decision-making processes, and consumption patterns of individuals, communities, and organizations (Dietz et al., 2009; Steg and Vlek, 2009). Human behavior is a primary driver of climate change, contributing to rising temperatures, ecosystem destruction, biodiversity loss, and extreme weather events such as floods, droughts, and wildfires (Abbass et al., 2022; Clarke et al., 2022). Consequently, promoting public pro-environmental behavior represents a vital approach to resolving environmental issues (Nielsen et al., 2021). Achieving positive environmental change requires behavioral modification, and understanding the relationships between different behaviors can facilitate this change. For example, individuals who drive electric vehicles may be more inclined to install solar panels (Ciocîrlan et al., 2025). Therefore, research on environmental issues must transcend the “behavioral isolation” mindset and focus on the potential causal links between behaviors, known as “spillover effects” (Galizzi and Whitmarsh, 2019). Recognizing the interconnectedness of pro-environmental behaviors enables researchers to develop comprehensive strategies for promoting sustainable practices and designing interventions that target multiple behaviors simultaneously.

Recent studies in environmental psychology have increasingly focused on spillover effects, defined as the extent to which engaging in past pro-environmental behavior influences the likelihood of subsequent pro-environmental actions (Lanzini and Thøgersen, 2014; Maki et al., 2019; Wan and Chen, 2023). Spillover effects can be categorized into positive and negative types based on the direction of influence (Carrico, 2021; Gregersen et al., 2025). Positive spillover occurs when one pro-environmental behavior increases the likelihood of future pro-environmental actions (Henn et al., 2020; Xu et al., 2018), whereas negative spillover occurs when one behavior reduces the likelihood of future actions (Jin et al., 2022; Tiefenbeck et al., 2013). The literature reports inconsistent findings regarding spillover effects. Many studies have documented positive spillover; for instance, changes in recycling behavior often coincide with a reduction in over-packaging (Thøgersen, 1999), and energy-saving driving styles correlate with intentions to reduce meat consumption (Van der Werff et al., 2013a). Others have reported negative spillover, such as increased purchases of organic products correlating with reduced recycling behavior (Thøgersen and Ölander, 2003), or participation in green energy programs leading to increased subsequent energy usage (Jacobsen et al., 2012). A few studies found no spillover effects; for example, increased usage of reusable bags while shopping was unrelated to other pro-environmental behaviors (Poortinga et al., 2013). Regardless of their direction, spillover effects reflect the actual outcomes of behavioral interventions, making their study crucial for environmental education and behavioral intervention (Galizzi and Whitmarsh, 2019; Xu et al., 2018). From a policymaker’s perspective, spillover effects can alter a range of behaviors at a low cost without resorting to unpopular regulatory measures. For researchers, examining these effects allows for evaluating single behavior change strategies and exploring complex relationships between different pro-environmental behaviors.

With the development of digital information technology, human behavior increasingly occurs within virtual environments (Yee, 2006). Among these, the Metaverse constitutes a crucial virtual space in daily life. The term combines “meta,” meaning transcendence, and “universe,” referring to a three-dimensional (3D) virtual space (Jin et al., 2022). The Metaverse is a virtual world linked and created using technology, which maps and interacts with the real world, serving as a digital living space with a new social system (Liu and Bai, 2022). While existing research has validated spillover effects of pro-environmental behaviors such as low-carbon travel and green consumption, the maturation of virtual reality (VR) technology raises questions about how to encourage public pro-environmental behavior through immersive technologies in the Metaverse. Previous studies have identified VR and immersive technologies as promising tools for raising awareness of current environmental issues and promoting individual pro-environmental intentions (Deringer and Hanley, 2021; Scurati et al., 2021). Recent research indicates that virtual tree-planting behavior in the Metaverse promotes subsequent pro-environmental interest and attitudes toward the real natural environment by enhancing environmental self-identity (Lam et al., 2025). However, other studies suggest that engaging in pro-environmental behavior using a self-customized avatar in the Metaverse can lead to a licensing effect, thereby reducing real-world pro-environmental behavior intentions (Jin et al., 2022). Consequently, it remains unclear whether the spillover effect of pro-environmental behavior in the Metaverse on real-world pro-environmental behavior is positive, negative, or a combination of both. Furthermore, the underlying mediating mechanisms have not been systematically explored. This study aims to systematically examine the spillover effects of pro-environmental behavior in the Metaverse on real-world pro-environmental behavior and its internal psychological mechanisms.

Theoretically, adopting environmentally friendly behaviors can generate positive spillover by enhancing environmental self-identity or negative spillover by alleviating negative emotions, such as guilt (Lacasse, 2016; Truelove et al., 2021). Environmental self-identity refers to the extent to which an individual considers themselves an environmentalist (Whitmarsh and O’Neill, 2010). According to self-perception theory, individuals understand themselves by observing the implications of their own behaviors, just as they understand others (Bem, 1972). Thus, engaging in pro-environmental behavior may lead people to perceive themselves as “environmentalists” or “green.” When individuals recognize their past environmental actions, they may exhibit a stronger environmental self-identity (Van der Werff et al., 2014a) and a greater moral obligation to continue such actions (Van der Werff et al., 2013a). Therefore, research suggests that environmental self-identity can serve as a mediator for the positive spillover of pro-environmental behavior. For instance, recalling past pro-environmental behaviors can strengthen individual environmental self-identity (Cornelissen et al., 2008; Lacasse, 2016; Van der Werff et al., 2014b), which correlates with increased green product choices (Van der Werff et al., 2014b), climate change concern, policy support (Lacasse, 2016), and pro-environmental intentions (Lauren et al., 2019; Truelove et al., 2016). Guilt is a negative emotion that arises when beliefs about acceptable behavior are violated (Jin and Oh, 2025; Shipley and Van Riper, 2022). Research has found that guilt can predict immediate pro-environmental behavior performance but fails to predict performance 2.5 h later (Bissing-Olson et al., 2016). If initial pro-environmental behavior is motivated by guilt, it may lead to negative spillover (Lacasse, 2016). The moral licensing effect explains this phenomenon (Truelove et al., 2021); when recent moral behavior enhances an individual’s moral self-image, they may feel less obligated to engage in further moral actions (Zhong et al., 2009). This theory suggests that individuals who have previously engaged in pro-environmental behavior may enhance their moral self-image, thereby reducing their sense of moral obligation and guilt (Truelove et al., 2014). Consequently, this may lead to a decreased willingness to continue practicing pro-environmental behavior in the future (Truelove et al., 2016). Empirical research also supports the notion that the alleviation of guilt is a pathway for negative spillover. For instance, Lacasse (2016) found that participants who perceived themselves as performing many pro-environmental behaviors reported greater environmental self-identity, which strengthened their environmental attitudes. Simultaneously, however, they reported reduced guilt, which weakened their environmental attitudes.

As mentioned above, pro-environmental behavior in the Metaverse may promote subsequent real-world pro-environmental interests and attitudes by enhancing individual environmental self-identity (Lam et al., 2025), or it may lead to a licensing effect, thereby reducing real-world pro-environmental behavior intentions (Jin et al., 2022). However, these two studies investigated the positive spillover or negative spillover of Metaverse pro-environmental behavior in isolation, which may explain the inconsistent results. Furthermore, most existing studies rely on self-reported pro-environmental intention measures, whereas research shows that intention does not necessarily represent actual pro-environmental behavior (Kollmuss and Agyeman, 2002). Regardless of whether spillover effects are positive or negative, they significantly influence subsequent environmental behavior. A thorough exploration of spillover effects can enhance our understanding of behavioral causes and effects, broadening research inquiries from specific intervention effects to the overall impact of spillover on non-specific behaviors. This enables a more comprehensive and realistic assessment of the ultimate effectiveness of interventions (Nilsson et al., 2017). As technology advances and virtual worlds gain popularity, systematically exploring the spillover effects of Metaverse pro-environmental behavior is crucial for understanding how to effectively integrate virtual reality into education and intervention programs to foster a sustainable future (Lam et al., 2025). Accordingly, this study aims to examine the spillover effects of pro-environmental behavior in the Metaverse on actual real-world pro-environmental behavior and its potential dual mediating mechanisms. The following hypotheses are proposed:

*H1a*: Participants engaging in pro-environmental behavior in the Metaverse may exhibit stronger environmental self-identity, thereby promoting subsequent real-world pro-environmental behavior (positive spillover pathway).

*H1b*: Participants engaging in pro-environmental behavior in the Metaverse may report lower levels of guilt, thereby reducing subsequent real-world pro-environmental behavior (negative spillover pathway).

*H1c*: Due to the simultaneous activation of both positive and negative spillover pathways, the total spillover effect from Metaverse pro-environmental behavior to real-world pro-environmental behavior may be insignificant.

Given the co-occurrence of positive and negative spillovers, identifying effective strategies to foster positive spillover effects while mitigating negative spillover merits further investigation (Geng et al., 2019; Lacasse, 2016). Research indicates that applying a social label of “environmentalist” to individuals who perceive themselves as having performed many pro-environmental behaviors enhances their environmental self-identity without reducing guilt, resulting in a total positive spillover effect from prior pro-environmental behavior to subsequent pro-environmental intentions (Lacasse, 2016). This phenomenon may stem from the premise that if individuals accept the labels assigned to them, their behavior tends to align with the implications of those labels. Social labeling involves the use of socially significant descriptors (e.g., “deviant,” “compassionate,” or “green”) to characterize an individual’s behavior, preferences, or values (Cornelissen et al., 2007; Eby et al., 2019). Similarly, applying the “environmentalist” label to individuals directly reinforces their environmental self-identity (Van der Werff et al., 2014b). Studies demonstrate that characterizing individuals as “environmentalists” enhances environmental self-identity, thereby preventing negative spillover effects while facilitating positive spillover effects (Geng et al., 2019). Recent findings further suggest that “environmentalist” labeling can bolster consumers’ environmental self-identity, which is positively correlated with the willingness to purchase pro-environmental products and engage in environmental behaviors, as well as with anticipated guilt (Jin and Oh, 2025). Furthermore, scholars propose that when individuals view themselves as “environmentalists,” they are less susceptible to the moral licensing effect; consequently, the influence of guilt alleviation on the spillover effects of pro-environmental behavior may be negated (Lacasse, 2016; Xu et al., 2018). Therefore, both theoretically and empirically, “environmentalist” labeling appears to be a crucial strategy for fostering positive—rather than negative—spillover effects.

However, it remains unclear whether “environmentalist” labeling can serve as an effective intervention for the spillover effects of pro-environmental behavior in the Metaverse. With advancements in virtual reality and immersive technologies, more implicit labeling strategies may emerge, and consumers in the virtual online world may similarly be subject to various personalized labels (Jin and Oh, 2025; Schwartz et al., 2020). Based on this, the present study further explores the role of “environmentalist” labeling as an intervention in the spillover effects of Metaverse pro-environmental behavior on real-world pro-environmental behavior. The following hypotheses are proposed:

*H2a*: Participants labeled as “environmentalists” after engaging in pro-environmental behavior in the Metaverse may continue to exhibit stronger environmental self-identity, thereby promoting subsequent real-world pro-environmental behavior (positive spillover pathway).

*H2b*: Participants labeled as “environmentalists” after engaging in pro-environmental behavior in the Metaverse may not exhibit reduced guilt, thereby blocking the negative spillover pathway.

*H2c*: The total spillover effect from Metaverse pro-environmental behavior to real-world pro-environmental behavior is expected to be positive.

In summary, this study aims to systematically explore the spillover effects of pro-environmental behavior in the Metaverse on real-world pro-environmental behavior and the underlying psychological mechanisms, while further seeking intervention strategies to foster positive spillover. Two studies were conducted to systematically test the proposed hypotheses. Study 1 preliminarily examines the spillover effects and mediating mechanisms of pro-environmental behavior in the Metaverse on real-world behavior to verify H1a, H1b, and H1c. Study 2 further investigates the intervention role of “environmentalist” labeling in the spillover effects of Metaverse pro-environmental behavior on real-world pro-environmental behavior to verify H2a, H2b, and H2c. Theoretically, this study aims to elucidate the complex psychological mechanisms by which behaviors in the Metaverse influence real-world actions and to reconcile the debates regarding inconsistent spillover directions in existing research. Practically, it aims to provide empirical support and scientific recommendations for utilizing Metaverse technology in environmental education and designing more effective persuasion strategies to facilitate the public’s transition toward sustainable lifestyles.

## 2 Study 1

### 2.1 Purpose and hypotheses

Study 1 aimed to examine the spillover effects of pro-environmental behavior in the Metaverse on real-world pro-environmental behavior, as well as the mediating roles of environmental self-identity and guilt. This study tested hypotheses H1a, H1b, and H1c.

### 2.2 Methods

#### 2.2.1 Participants

Following previous research (Jin et al., 2022; Lacasse, 2016), this study adopted a single-factor, two-level between-subjects design. The sample size was determined based on similar designs used by Jin et al. (*N* = 60) and Study 1 by Lacasse (*N* = 120). Accordingly, 134 undergraduate and graduate students were recruited on a voluntary basis from a comprehensive university in China, representing both social science and science or technology disciplines. Six participants were excluded from the statistical analysis due to failure to engage attentively with the Metaverse platform tasks or excessively short self-reporting times. Ultimately, 128 participants were included in the analysis (*M* ± *SD* = 20.52 ± 2.20 years; 108 females, 20 males). All participants signed an informed consent form and were compensated upon completion of the study. Participants were randomly assigned to two groups: the Metaverse Pro-environmental Group (*N* = 67) and the Control Group (*N* = 61). A sensitivity power analysis indicated that this sample size was sufficient to detect an effect size of *d* = 0.50 with a statistical power of 0.80 at a significance level of α = 0.05 (two-tailed) (G*Power 3.1) (Faul et al., 2007).

#### 2.2.2 Design

A single-factor, two-level between-subjects design was employed (Metaverse pro-environmental behavior: Pro-environmental Group vs. Control Group).

#### 2.2.3 Materials

##### 2.2.3.1 Metaverse platform

ZEPETO is a free mobile application offering augmented reality (AR), 3D avatars, and social networking services. Launched globally in August 2018 across 165 markets, the platform has accumulated over 200 million users. Using facial recognition technology, users can create 3D avatars resembling themselves, interact with other users, and engage in diverse virtual reality experiences in official ZEPETO worlds or user-generated scenarios. Following previous research (Jin et al., 2022), a virtual café named “GREEN CAFE” was created in ZEPETO using the “ZEPETO Build It” program. The first floor was designed to resemble a realistic café, while the second floor served as a pro-social exhibition space, decorated with numerous posters promoting pro-environmental behaviors.

##### 2.2.3.2 Metaverse pro-environmental behavior

Adapting the method from Jin et al. (2022), the virtual “GREEN CAFE” in the Metaverse platform ZEPETO served as the experimental setting. The first floor functioned as a standard café, while the second floor was a poster exhibition hall featuring themes such as “Green Living,” “Reducing Single-use Plastic Pollution,” “Guarding the Ocean,” and “Protecting Forests.” Both the Pro-environmental Group and the Control Group received instructions on how to operate their virtual avatars. After mastering the controls, participants were required to complete an online game task.

In the online game task, participants in the Pro-environmental Group were informed that the task was a Metaverse pro-environmental behavior mission. They were required to walk around the café, proceed to the second floor to read the posters, and were informed at the end of the task that the experimenters would donate 10 CNY on their behalf to the “Tencent Charity: Western Desert Tree Planting Environmental Fundraising Project” (i.e., engaging in a pro-environmental donation). Conversely, participants in the Control Group were informed that the task was a visitation mission. They were simply required to walk around the café and proceed to the second floor to read the posters; no information regarding donations was provided.

##### 2.2.3.3 Environmental self-identity scale

Based on previous studies (Van der Werff et al., 2013a,b), the Environmental Self-identity Scale consisted of three items (e.g., “Acting environmentally friendly is an important part of who I am,” “I am the type of person who acts environmentally friendly,” “I see myself as an environmentally friendly person”). Participants rated each item on a 10-point Likert scale (1 = Strongly Disagree, 10 = Strongly Agree). In this study, the Cronbach’s α coefficient for this scale was 0.84.

##### 2.2.3.4 Guilt scale

Based on previous research (Onwezen et al., 2014), the Guilt Scale comprised three items (“I feel guilty,” “I feel remorseful,” “I have a bad conscience”). Participants rated each item on a 10-point Likert scale (1 = Do not experience this emotion at all, 10 = Experience this emotion very strongly). In this study, the Cronbach’s α coefficient for this scale was 0.95.

##### 2.2.3.5 Real-world pro-environmental behavior

Following Zaval et al. (2015), an “Ecological Donation Task” was employed to measure real-world pro-environmental behavior. The actual amount donated by participants in this task served as the indicator. The specific content presented to participants was as follows:

*Charity Fundraiser | Kidney of the Earth: Clean Beach Action | Tencent Charity.*

Wetlands are known as the “Kidneys of the Earth.” The World Conservation Strategy clearly states that wetlands, along with forests and oceans, constitute the three major global ecosystems, playing an indispensable role in maintaining the Earth’s ecological balance. As the landscape with the richest biodiversity, wetlands are closely linked to human survival, reproduction, and development. However, due to the proximity of the Dongzhaigang Nature Reserve to densely populated areas and floating marine debris, coastal waste has not been cleaned up in time. This waste, primarily consisting of non-degradable plastics and packaging wrappers that are difficult to clean on a large scale, has caused the death of birds and other animals in the reserve, seriously affecting the natural ecology of Dongzhaigang. The Dongzhaigang Mangrove Reserve is in urgent need of a scientific, normalized, effective, and society-wide garbage cleanup campaign. Therefore, we plan to launch the “Clean Beach Plan” for the protection of the Dongzhaigang Mangrove Wetland to clean up garbage within and around the reserve in a timely, normalized, and scientific manner. Through charity, we aim to restore a clean home for the animals and plants we live with.

In this experiment, you will receive an initial compensation of 20 CNY. Are you willing to donate a portion of your compensation to the “Clean Beach Plan” launched by environmental charity organizations? Your final actual compensation will be 20 CNY minus the donation amount. How much are you willing to donate? (Please enter the amount you wish to donate).

#### 2.2.4 Procedure

Prior to the formal experiment, participants were randomly assigned to either the Pro-environmental Group or the Control Group and completed the informed consent form.

At the start of the experiment, participants received instructions regarding the ZEPETO application. Before entering the Metaverse, participants created a virtual avatar based on a selfie and were allowed to customize the avatar’s appearance and clothing. Subsequently, participants were guided into the ZEPETO Metaverse, received instructions on operating the avatar and the online game task, and were asked to walk around the “GREEN CAFE.” They were then required to move to the second floor and carefully read the environmental awareness posters. Finally, the Pro-environmental Group was informed that this was a Metaverse pro-environmental behavior task and that 10 CNY would be donated on their behalf to the “Tencent Charity: Western Desert Tree Planting Environmental Fundraising Project.” The Control Group was told that this was a visitation task without being informed of any donation-related information.

**FIGURE 1:**
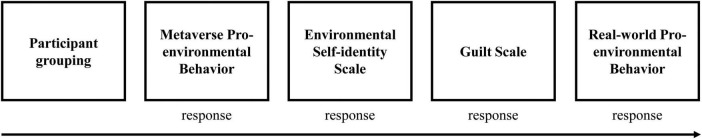
Flowchart of STUDY 1.

After completing the online Metaverse task, participants immediately exited the platform and subsequently completed the Environmental Self-identity Scale, the Guilt Scale, and the “Ecological Donation Task” offline. Upon conclusion, participants received their corresponding remuneration (i.e., 20 CNY initial fee minus the actual donation amount). The procedure is illustrated in Figure 1.

#### 2.2.5 Data statistics and analysis

Firstly, regression analyses using SPSS 29.0 revealed that neither gender nor age significantly predicted real-world pro-environmental behavior (*p*s > 0.05). Subsequently, after controlling for gender, age, and education level, the main effect of Metaverse pro-environmental behavior (Pro-environmental Group vs. Control Group) was tested using regression analysis in Mplus 8.3. Finally, a path model was constructed using Mplus 8.3 to explore the mediating roles of environmental self-identity and guilt in the effect of Metaverse pro-environmental behavior on real-world pro-environmental behavior (pro-environmental donation). Bias-corrected bootstrap confidence intervals (95% CI) were calculated based on 5,000 bootstrap samples. Additionally, collinearity checks revealed that Variance Inflation Factors (VIF) were all less than 4, indicating no serious multicollinearity issues in this study (Dormann et al., 2013).

### 2.3 Results

#### 2.3.1 Descriptive statistics and main effects test

Table 1 presents the descriptive statistics and the results of the main effects test for Metaverse pro-environmental behavior. The results indicated that the effect of Metaverse pro-environmental behavior on real-world pro-environmental behavior (pro-environmental donation) was not significant [*t* = 0.679, *p* = 0.497, 95% CI = (–0.112, 0.232)]. Specifically, there was no significant difference in donation amounts between the Pro-environmental Group (*M* ± *SD* = 8.40 ± 5.43) and the Control Group (*M* ± *SD* = 7.77 ± 5.72). However, Metaverse pro-environmental behavior significantly and positively influenced environmental self-identity [*t* = 4.144, *p* < 0.001, 95% CI = (0.171, 0.479)]; the environmental self-identity of the Pro-environmental Group (*M* ± *SD* = 8.23 ± 1.34) was significantly higher than that of the Control Group (*M* ± *SD* = 7.05 ± 2.11). Furthermore, Metaverse pro-environmental behavior significantly and negatively influenced guilt [*t* = –2.466, *p* = 0.014, 95% CI = (–0.374, –0.043)]; the guilt reported by the Pro-environmental Group (*M* ± *SD* = 2.45 ± 2.06) was significantly lower than that of the Control Group (*M* ± *SD* = 3.24 ± 1.88).

**TABLE 1:** Descriptive statistics and main effects test results for Study 1.

Results	Pro-environmental group (*N* = 67)	Control group (*N* = 61)	*t*	*p*	95% CI
	*M* (*SD*)	*M* (*SD*)			
Pro-environmental donation	8.40 (5.43)	7.77 (5.72)	0.679	0.497	[–0.112, 0.232]
Environmental self-identity	8.23 (1.34)	7.05 (2.11)	4.144	< 0.001	[0.171, 0.479]
Guilt	2.45 (2.06)	3.24 (1.88)	–2.466	0.014	[–0.374, –0.043]

#### 2.3.2 Mediation model analysis

**FIGURE 2:**
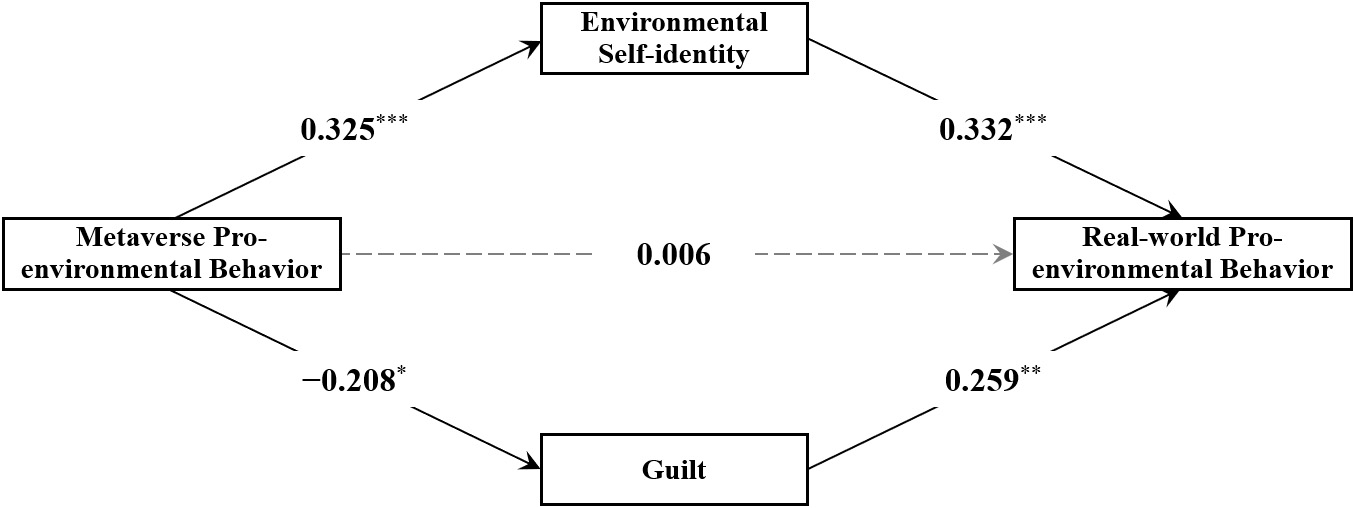
Analysis results of the mediation model in Study 1 (standardized results). *p < 0.05, **p < 0.01, ***p < 0.001.

The mediation model analysis revealed that while the direct effect of Metaverse pro-environmental behavior on real-world pro-environmental behavior (pro-environmental donation) was not significant [β = 0.006, *p* = 0.956, 95% CI = (–0.188, 0.197)], it had a significant positive effect on environmental self-identity [β = 0.325, *p* < 0.001, 95% CI = (0.161, 0.466)] and a significant negative effect on guilt [β = –0.208, *p* = 0.015, 95% CI = (–0.368, -0.035)]. Furthermore, both environmental self-identity [β = 0.332, *p* < 0.001, 95% CI = (0.147, 0.505)] and guilt [β = 0.259, *p* = 0.001, 95% CI = (0.092, 0.411)] significantly and positively influenced real-world pro-environmental behavior (see Figure 2).

Subsequently, the Bootstrap method (5,000 samples) was used to calculate the mediating effects of environmental self-identity and guilt. The results showed that the 95% confidence interval for the mediating effect of environmental self-identity in the relationship between Metaverse pro-environmental behavior and real-world pro-environmental behavior did not include 0, indicating a significant mediating effect. Similarly, the 95% confidence interval for the mediating effect of guilt in the same relationship did not include 0, indicating that this mediating effect was also significant (see Table 2).

**TABLE 2:** Mediation effect results for Study 1 (standardized results).

Path	β	*SE*	95% CI
Metaverse pro-environmental behavior → environmental self-identity → real-world pro-environmental behavior	0.108	0.039	[0.045, 0.204]
Metaverse pro-environmental behavior → guilt → real-world pro-environmental behavior	–0.054	0.027	[–0.119, –0.013]

### 2.4 Discussion

Study 1 found that engaging in pro-environmental behavior in the Metaverse (prior pro-environmental donation) directly influenced individuals’ environmental self-identity and guilt, and indirectly affected their real-world pro-environmental behavior (subsequent pro-environmental donation) through these two mediators. This suggests that when individuals engage in pro-environmental behavior in the virtual world, both positive spillover and negative spillover processes occur. When people realize they have performed an act beneficial to the environment, even within a virtual setting (Lam et al., 2025), it leads them to perceive themselves as environmentally responsible, thereby enhancing environmental self-identity (Cornelissen et al., 2008; Van der Werff et al., 2014b). This enhanced identity fosters behavioral consistency and promotes subsequent real-world pro-environmental behaviors (supporting the positive spillover pathway and H1a). However, this pro-environmental behavior originating from the virtual world also reduced individuals’ guilt (Jin et al., 2022). This reduction may stem from the realization that they have already performed an environmental action, leading to a moral licensing effect (Truelove et al., 2014) and a further alleviation of guilt (Truelove et al., 2016), which consequently resulted in a reduction of subsequent pro-environmental behaviors (supporting the negative spillover pathway and H1b). As both positive and negative spillover pathways were active simultaneously, the total spillover effect from the Metaverse to the real world was not significant (supporting H1c).

Study 2 aims to enhance the positive spillover pathway while attenuating the negative spillover pathway. By applying the “environmentalist” label to participants who perform pro-environmental behaviors in the Metaverse, we aim to guide them to further strengthen their environmental self-identity (Geng et al., 2019; Jin and Oh, 2025) and interpret their past pro-environmental behaviors as a reflection of this identity. Thus, Study 2 verifies whether “environmentalist” labeling can further augment the positive spillover of Metaverse pro-environmental behavior to real-world pro-environmental behavior. Moreover, when individuals view themselves as “environmentalists,” they are less susceptible to the moral licensing effect, and the influence of guilt alleviation on spillover effects may be negated (Lacasse, 2016; Xu et al., 2018). Study 2 aims to further verify whether “environmentalist” labeling can weaken the negative spillover of Metaverse pro-environmental behavior. Ultimately, the enhancement of positive spillover combined with the attenuation of negative spillover may cause the total spillover effect from Metaverse pro-environmental behavior to real-world pro-environmental behavior to shift towards a positive outcome.

## 3 Study 2

### 3.1 Purpose and hypotheses

Study 2 aimed to further examine the intervention effect of “environmentalist” labeling on the spillover effects from Metaverse pro-environmental behavior to real-world pro-environmental behavior, thereby testing hypotheses H2a, H2b, and H2c.

### 3.2 Methods

#### 3.2.1 Participants

Following previous research (Jin et al., 2022; Lacasse, 2016), this study adopted a single-factor, two-level between-subjects design, identical to the design used by Jin et al. (total *N* = 60) and Study 2 by Lacasse (total *N* = 67). Accordingly, 80 undergraduate and graduate students were recruited on a voluntary basis from a comprehensive university in China, representing both social science and science or technology disciplines. Ten participants were excluded from the statistical analysis due to failure to engage diligently with the Metaverse platform tasks or for having excessively short self-reporting times. Ultimately, 70 participants were included in the analysis (*M* ± *SD* = 20.80 ± 5.26 years; 58 females, 12 males). All participants signed an informed consent form and were compensated upon completion of the study. Participants were randomly assigned to two groups: the Metaverse Pro-environmental + Labeling Group (*N* = 37) and the Control Group (*N* = 33). A sensitivity power analysis indicated that this sample size was sufficient to detect an effect size of *d* = 0.68 with a statistical power of 0.80 at a significance level of α = 0.05 (two-tailed) (G*Power 3.1) (Faul et al., 2007).

#### 3.2.2 Design

A single-factor, two-level between-subjects design was employed (Metaverse pro-environmental behavior: Pro-environmental + Labeling Group vs. Control Group). Specifically, participants in the Pro-environmental + Labeling Group were assigned the “environmentalist” label after completing the Metaverse pro-environmental behavior task, whereas participants in the Control Group were not assigned the “environmentalist” label after completing the visitation task.

#### 3.2.3 Materials

##### 3.2.3.1 Metaverse platform

Consistent with Study 1, ZEPETO was used as the Metaverse platform.

##### 3.2.3.2 Metaverse pro-environmental behavior

The procedure was consistent with Study 1. Participants in the Pro-environmental + Labeling Group were required to complete a Metaverse pro-environmental behavior task by engaging in activities within the virtual “GREEN CAFE” in ZEPETO according to instructions. At the end of the task, they were informed that the experimenters would donate 10 CNY on their behalf to the “Tencent Charity: Western Desert Tree Planting Environmental Fundraising Project” (i.e., engaging in a pro-environmental donation). Participants in the Control Group were required to complete a visitation task by engaging in activities within the virtual “GREEN CAFE” according to instructions, without being informed of any donation-related information.

##### 3.2.3.3 “Environmentalist” labeling

Following previous studies (Geng et al., 2019; Lacasse, 2016), a “labeling” manipulation was employed. For the Pro-environmental + Labeling Group, a labeling procedure was administered immediately after they completed the Metaverse pro-environmental behavior task. They received a small card displaying a green promotional message stating: “*You start with small daily actions, care for the environment, conserve energy, and have contributed to mitigating the greenhouse effect. You are an environmentalist!*” This statement explicitly labeled them as environmentalists. Conversely, after completing the visitation task, the Control Group received a small card displaying a more common green promotional message stating: “*We should all start with small daily actions, care for the environment, conserve energy, and contribute our strength to mitigating the greenhouse effect. We should all strive to become environmentalists!*” This statement served as a general exhortation rather than a label.

##### 3.2.3.4 Environmental self-identity scale

Consistent with Study 1.

##### 3.2.3.5 Guilt scale

Consistent with Study 1.

##### 3.2.3.6 Real-world pro-environmental behavior

Consistent with Study 1.

#### 3.2.4 Procedure

Prior to the formal experiment, participants were randomly assigned to either the Pro-environmental + Labeling Group or the Control Group and completed the informed consent form.

At the start of the experiment, participants received instructions regarding the ZEPETO application. Before entering the ZEPETO Metaverse, participants created a virtual avatar based on a selfie and were allowed to customize the avatar’s appearance and clothing. Subsequently, participants were guided into the ZEPETO Metaverse and received instructions on operating the avatar and the online game task, consistent with Study 1. Finally, participants in the Pro-environmental + Labeling Group were informed upon completion of the Metaverse pro-environmental behavior task that the experimenters would donate 10 CNY on their behalf to the “Tencent Charity: Western Desert Tree Planting Environmental Fundraising Project.” They then received the small card with the green promotional message containing the specific declaration, “You are an environmentalist.” For the Control Group, upon completion of the visitation task, no donation information was provided. They subsequently received the small card with the green promotional message containing the general declaration, “We should all strive to become environmentalists.”

After completing the online Metaverse task, participants immediately exited the platform and subsequently completed the Environmental Self-identity Scale, the Guilt Scale, and the “Ecological Donation Task” offline. Upon conclusion, participants received their corresponding remuneration (i.e., 20 CNY initial fee minus the actual donation amount). Therefore, the procedures for Study 2 and Study 1 are largely similar, with the primary distinction being that participants in Study 2 were assigned the “Environmentalist” Labeling or not during the online Metaverse task.

#### 3.2.5 Data statistics and analysis

First, regression analyses using SPSS 29.0 revealed that neither gender nor age significantly predicted real-world pro-environmental behavior (*p*s > 0.05). Second, after controlling for gender, age, and education level, the main effect of the Metaverse pro-environmental behavior combined with “environmentalist” labeling manipulation was tested using regression analysis in Mplus 8.3. Finally, a path model was constructed using Mplus 8.3 to explore the mediating roles of environmental self-identity and guilt in the effect of the manipulation on real-world pro-environmental behavior (pro-environmental donation). Bias-corrected bootstrap confidence intervals (95% CI) were calculated based on 5,000 bootstrap samples. Additionally, collinearity checks revealed that Variance Inflation Factors (VIF) were all less than 4, indicating no serious multicollinearity issues in this study (Dormann et al., 2013).

### 3.3 Results

#### 3.3.1 Descriptive statistics and main effects test

Table 3 presents the descriptive statistics and the results of the main effects test for the Metaverse pro-environmental behavior combined with “environmentalist” labeling manipulation. The results indicated that the manipulation significantly and positively influenced real-world pro-environmental behavior (pro-environmental donation) [*t* = 5.060, *p* < 0.001, 95% CI = (0.293, 0.663)]. Specifically, the donation amount in the Pro-environmental + Labeling Group (*M* ± *SD* = 10.27 ± 6.76) was significantly higher than that in the Control Group (*M* ± *SD* = 4.30 ± 3.99). Furthermore, the manipulation significantly and positively influenced environmental self-identity [*t* = 2.972, *p* = 0.003, 95% CI = (0.108, 0.527)]; the environmental self-identity in the Pro-environmental + Labeling Group (*M* ± *SD* = 8.25 ± 1.42) was significantly higher than that in the Control Group (*M* ± *SD* = 7.26 ± 1.53). However, the effect of the manipulation on guilt was not significant [*t* = –1.550, *p* = 0.121, 95% CI = (–0.401, 0.047)], indicating no significant difference in guilt between the Pro-environmental + Labeling Group (*M* ± *SD* = 1.91 ± 1.79) and the Control Group (*M* ± *SD* = 2.60 ± 1.88).

**TABLE 3:** Descriptive statistics and main effects test results for Study 2.

Results	Pro-environmental + labeling group (*N* = 37)	Control group (*N* = 33)	*t*	*p*	95% CI
	*M* (*SD*)	*M* (*SD*)			
Pro-environmental donation	10.27 (6.76)	4.30 (3.99)	5.060	< 0.001	[0.293, 0.663]
Environmental self-identity	8.25 (1.42)	7.26 (1.53)	2.972	0.003	[0.108, 0.527]
Guilt	1.91 (1.79)	2.60 (1.88)	–1.550	0.121	[–0.401, 0.047]

#### 3.3.2 Mediation model analysis

**FIGURE 3:**
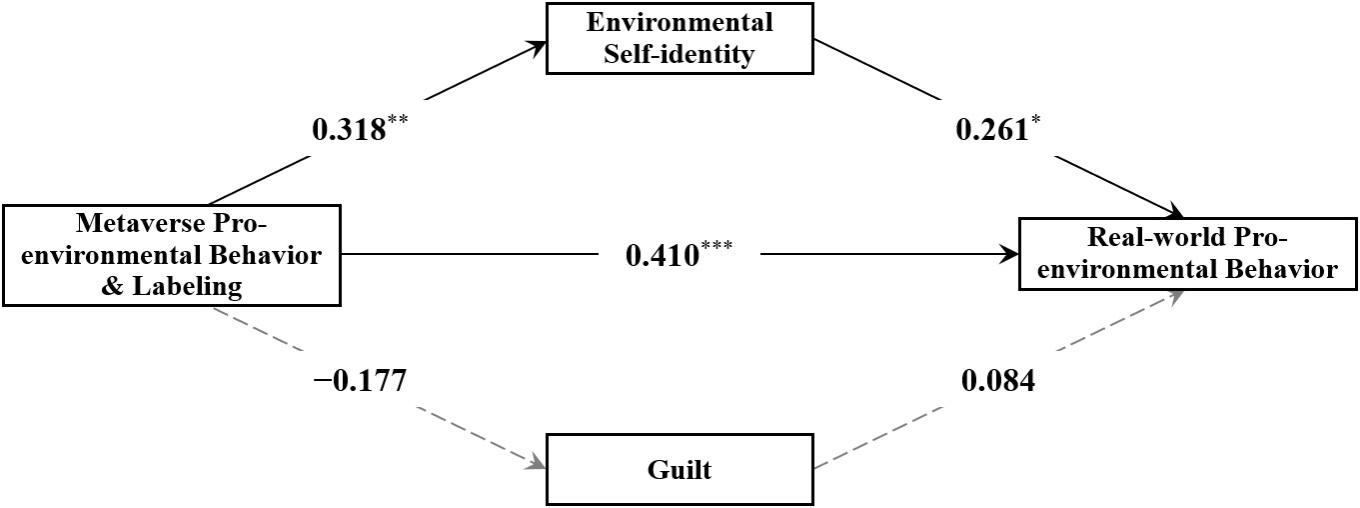
Analysis results of the mediation model in Study 2 (standardized results). *p < 0.05, **p < 0.01, ***p < 0.001.

The mediation model analysis revealed that the Metaverse pro-environmental behavior combined with “environmentalist” labeling manipulation had a significant positive effect on real-world pro-environmental behavior [β = 0.410, *p* < 0.001, 95% CI = (0.203, 0.580)] and a significant positive effect on environmental self-identity [β = 0.318, *p* = 0.003, 95% CI = (0.104, 0.521)]. However, its effect on guilt was not significant [β = –0.177, *p* = 0.159, 95% CI = (–0.416, 0.072)]. Additionally, environmental self-identity significantly and positively influenced real-world pro-environmental behavior [β = 0.261, *p* = 0.014, 95% CI = (0.033, 0.456)], whereas the effect of guilt on real-world pro-environmental behavior was not significant [β = 0.084, *p* = 0.578, 95% CI = (–0.169, 0.422)] (see Figure 3).

Subsequently, the Bootstrap method (5,000 samples) was used to calculate the mediating effects of environmental self-identity and guilt. The results showed that the 95% confidence interval for the mediating effect of environmental self-identity in the relationship between the manipulation and real-world pro-environmental behavior did not include 0, indicating a significant mediating effect. However, the 95% confidence interval for the mediating effect of guilt included 0, indicating that the mediating effect of guilt was not significant (see Table 4).

**TABLE 4:** Mediation effect results for Study 2 (standardized results).

Path	β	*SE*	95% CI
Metaverse pro-environmental behavior and labeling → environmental self-identity → real-world pro-environmental behavior	0.083	0.044	[0.015, 0.200]
Metaverse pro-environmental behavior and labeling → guilt → real-world pro-environmental behavior	–0.015	0.035	[–0.122, 0.026]

### 3.4 Discussion

Study 2 found that adding “environmentalist” labeling to the prior Metaverse pro-environmental behavior manipulation altered its spillover effects. When participants were labeled as “environmentalists” after performing pro-environmental behavior in the virtual Metaverse world, they were motivated to live up to this expectation (Eby et al., 2019; Schwartz et al., 2020). This led to an enhancement of their environmental self-identity (Geng et al., 2019; Van der Werff et al., 2014b), which in turn promoted their subsequent real-world pro-environmental behavior (confirming the positive spillover pathway and H2a).

Furthermore, the effect of reduced guilt—typically caused by perceiving oneself as having already acted pro-environmentally—ceased to exist. The “environmentalist” label carries substantial behavioral and attitudinal expectations (Cornelissen et al., 2007; Lacasse, 2016). This label likely conveys to individuals that they should perform even more actions and reminds them that there is still a long way to go to truly meet this standard (Jin and Oh, 2025; Xu et al., 2018). Consequently, after being labeled as “environmentalists,” participants’ guilt did not decrease significantly and may have continued to motivate them at a level similar to the Control Group (the negative spillover pathway disappeared, confirming H2b). As the positive spillover pathway was activated while the negative spillover pathway was effectively blocked, the total spillover effect from Metaverse pro-environmental behavior to real-world pro-environmental behavior shifted to a positive spillover (confirming H2c).

## 4 General discussion

### 4.1 Spillover effects of pro-environmental behavior in the Metaverse: the mediating roles of environmental self-identity and guilt

The results of this study indicate that engaging individuals in pro-environmental behavior within the Metaverse systematically influences the likelihood of their subsequent real-world pro-environmental behavior. This provides empirical evidence for the spillover of pro-environmental behavior from the virtual Metaverse to the real world. Specifically, individuals who engage in pro-environmental behavior in the Metaverse exhibit higher levels of environmental self-identity, which further promotes their subsequent real-world pro-environmental behavior, demonstrating a positive spillover effect. According to self-perception theory, people come to know themselves by observing their own behavior (Bem, 1972). Thus, performing pro-environmental actions may lead individuals to perceive themselves as environmentally friendly. Environmental self-identity represents the extent to which individuals view themselves as environmentalists (Whitmarsh and O’Neill, 2010). Consequently, when individuals recognize their past pro-environmental behaviors, their environmental self-identity is enhanced (Van der Werff et al., 2013a,2014a). Furthermore, the promoting effect of environmental self-identity on pro-environmental behavior has been widely verified (Lacasse, 2016; Lauren et al., 2019; Van der Werff et al., 2013b). Therefore, this study confirms the mediating pathway through which prior pro-environmental behavior in the Metaverse enhances subsequent real-world pro-environmental behavior via increased environmental self-identity. Conversely, individuals who engage in pro-environmental behavior in the Metaverse also exhibit lower levels of guilt, which may lead to a reduction in their subsequent real-world pro-environmental behavior, demonstrating a negative spillover effect. According to the moral licensing effect, an individual’s moral self-image is bolstered after performing an initial moral act, which may diminish their subsequent sense of moral obligation and reduce the likelihood of further moral actions (Zhong et al., 2009). Related research has shown that previous pro-environmental behavior performed by individuals may enhance their moral self-image, reduce their sense of guilt (Truelove et al., 2014), and thereby decrease their willingness to continue engaging in pro-environmental behavior (Truelove et al., 2016). Consequently, prior pro-environmental behavior may lead to a reduction in guilt. However, guilt is a key emotional factor driving pro-environmental behavior (Adams et al., 2020; Shipley and Van Riper, 2022). Thus, within the virtual Metaverse, this study verifies the mediating pathway through which prior pro-environmental behavior inhibits subsequent real-world pro-environmental behavior via reduced guilt.

Furthermore, as both positive spillover and negative spillover pathways are activated, the total spillover effect from Metaverse pro-environmental behavior to real-world pro-environmental behavior is not significant. This study addresses the limitations of previous research that examined spillover effects from virtual world behavior solely through a unidirectional pathway (Jin et al., 2022; Lam et al., 2025) and offers insights into the inconsistent results found in prior studies. For instance, Lam et al. (2025) found that pro-environmental behavior in the virtual world might enhance subsequent real-world pro-environmental interests and attitudes by strengthening individuals’ environmental self-identity. In contrast, Jin et al. (2022) reported that individuals might experience a licensing effect due to pro-environmental behavior performed in the Metaverse, leading to reduced pro-environmental intentions in reality. Some studies have even failed to find any spillover effects (Carrico et al., 2018); for example, an increased likelihood of bringing reusable bags while shopping was unrelated to other pro-environmental behaviors (Poortinga et al., 2013). The findings of this study indicate that the spillover pathways of pro-environmental behavior in the Metaverse are not unidirectional; rather, positive and negative spillover pathways may coexist. This partly explains the inconsistent results regarding unidirectional spillover pathways in previous literature. Moreover, most previous studies relied on self-reported measures of pro-environmental intentions, which do not necessarily represent actual pro-environmental behavior (de Matos et al., 2025; Kollmuss and Agyeman, 2002). This study employed a participant fee donation method to explore individuals’ actual pro-environmental behavior, assessing the practical effects of pro-environmental behavior spillover from a broader and more realistic perspective. This approach enhances the ecological validity and explanatory power of the findings. Most importantly, identifying pro-environmental behavior spillover effects within the Metaverse reveals the complex pathways through which virtual pro-environmental behavior influences real-world actions via dual psychological mechanisms (environmental self-identity and guilt). This provides key scientific evidence for designing effective Metaverse intervention strategies to promote sustainable behavior, contributing to emerging discussions regarding the role of the virtual Metaverse and VR technologies in environmental protection efforts.

### 4.2 The intervention effect of “environmentalist” labeling on the spillover effects of pro-environmental behavior in the Metaverse

This study further finds that when individuals recognize their pro-environmental behavior and are assigned the “environmentalist” label, this enhances the positive spillover effect by strengthening environmental self-identity and prevents the negative spillover effect by mitigating guilt. As a result, the total spillover effect from Metaverse pro-environmental behavior to real-world pro-environmental behavior shifts to a positive spillover. This demonstrates how the interpretation of past behavior influences self-perception and future attitudes and behaviors (Cornelissen et al., 2008). When individuals accept a label from others, it may create an expectation to behave consistently with that label (Burger and Caldwell, 2003). In the context of environmental labeling, individuals labeled as “environmentally conscious” after making eco-friendly choices are more likely to select other eco-friendly products in subsequent tasks (Cornelissen et al., 2007). Self-identity is often interpreted as a label people use to describe themselves (Cook et al., 2002). Therefore, applying the “environmentalist” label to individuals directly reinforces their environmental self-identity, that is, the extent to which individuals consider themselves environmentalists (Geng et al., 2019; Van der Werff et al., 2014b). Furthermore, the mechanism of reduced guilt may not function in this context because individuals who are more likely to view themselves as “environmentalists” are less susceptible to the moral licensing effect (Lacasse, 2016; Xu et al., 2018). Consequently, “environmentalist” labeling serves as a crucial method for creating positive spillover. These findings further support self-perception theory (Bem, 1972), as receiving the “environmentalist” label may cause individuals to confirm that they value the environment. This confirmation of their own behavior further deepens their self-cognition as “environmentalists,” verifying that identity perception is one of the theories explaining the persuasive impact of social labeling (Jin and Oh, 2025).

In addition to the critical role of environmental self-identity in pro-environmental behavior spillover effects, future research should also consider other variables, such as self-efficacy. For instance, Lauren et al. (2016) examined the role of self-efficacy in green behavior spillover, noting that self-efficacy gained from successfully engaging in green behavior can positively influence positive spillover. Similarly, Steinhorst et al. (2015) found that information interventions improved participants’ knowledge about climate change and skills to mitigate it, enhancing self-efficacy and facilitating positive spillover. Crucially, this study identifies the intervention effect of “environmentalist” labeling within the virtual Metaverse. Research suggests that labeling can serve as a social strategy to motivate behavioral change, as accepting a label can lead individuals to act in ways consistent with it (Cornelissen et al., 2007). To effectively engage the public, social marketers can design and implement “environmentalist” labeling strategies that align with the audience’s identity. With advancements in personalization technology, more implicit labeling strategies may emerge, allowing consumers to encounter more environmental advertisements and products on social media, which can help them identify with environmental values (Jin and Oh, 2025). This study confirms that assigning the “environmentalist” label to individuals who have performed pro-environmental behavior in the Metaverse can ensure positive spillover from virtual experiences to real-world pro-environmental behavior by reinforcing environmental self-identity and shielding against the moral licensing effect. This finding not only expands the theoretical understanding of how virtual identity construction impacts behavioral decision-making but also provides an effective intervention pathway for using digital social labeling strategies to cultivate public environmental habits in reality.

### 4.3 Limitations and future directions

This study systematically examines the spillover effects of pro-environmental behavior in the Metaverse on real-world pro-environmental behavior and its mediating mechanisms, while further exploring the intervention role of “environmentalist” labeling. Although the conclusions enrich theories such as self-perception theory and the moral licensing effect and provide important references for future exploration, certain limitations remain. Future research could improve and expand upon the following aspects:

First, in this study, participants in the Pro-environmental Group were informed that the experimenters would donate 10 CNY on their behalf (i.e., engaging in a pro-environmental donation). Therefore, these participants may have been additionally influenced by the social learning effect of example behavior, which could further affect their subsequent real-world pro-environmental donation behavior. Future research could exclude such social learning effects by either eliminating the experimenter’s donation behavior or incorporating other experimental outcomes that differ from the participants’ donation behavior, such as engaging participants in activities like waste sorting, eco-friendly crafts, or environmental advocacy. Additionally, the manipulation of “environmentalist” labeling and Metaverse pro-environmental behavior was not separated. Consequently, it was impossible to explore the independent effect of “environmentalist” labeling, making it difficult to distinguish whether the observed effects stemmed from the pro-environmental behavior itself, the reinforcement of the label, or the interaction between the two. Future research could employ a two-factor design to analyze the main effects and moderating roles of “environmentalist” labeling, thereby more precisely isolating and quantifying the independent contribution of labeling as a social intervention and its synergistic mechanism with prior pro-environmental behavior.

Second, the participants in this study were primarily university students. The homogeneity of the student sample limits the generalizability of the research conclusions, making it difficult to represent the behavioral responses of the broader public when facing complex trade-offs between cost and convenience in the real world. Future research could diversify the participant pool by including individuals of different ages, educational backgrounds, income levels, and cultural environments to test the universality and boundary conditions of the model and conclusions of this study. Additionally, there is an imbalance in the gender ratio of the sample, with a majority of female participants. Future research should aim to achieve a more balanced gender representation during participant recruitment.

Third, Study 1 indicated that Metaverse pro-environmental behavior influences subsequent real-world pro-environmental behavior through complete mediation by environmental self-identity and guilt, while Study 2 showed incomplete mediation. This may suggest that the “environmentalist” labeling in Study 2 has a strong facilitative effect on subsequent real-world pro-environmental behavior, leading to the discovery of a positive direct effect of the combined influence of Metaverse pro-environmental behavior and the “environmentalist” labeling on subsequent real-world pro-environmental behavior. However, this possibility still needs to be further validated in future research.

Finally, this study primarily employed behavioral methods and could not reveal the intrinsic neural mechanisms underlying the spillover effects of pro-environmental behavior in the Metaverse. Future research could combine Event-Related Potentials (ERP) technology, which has high temporal resolution, with Functional Magnetic Resonance Imaging (fMRI) technology, which has high spatial resolution. By systematically verifying the conclusions of this study from both temporal and spatial dimensions, future studies could further elucidate the underlying neurophysiological basis. Additionally, this study used laboratory tasks to assess pro-environmental behavior, which may not fully capture the complexity of such behavior in real-world situations. To enhance the ecological validity and generalizability of the research, future studies could employ more ecologically valid interpersonal interaction paradigms, field experiments, or observational studies.

## 5 Conclusion

(1) Individuals who engage in pro-environmental behavior in the Metaverse exhibit stronger environmental self-identity, which in turn promotes subsequent real-world pro-environmental behavior; simultaneously, they exhibit lower levels of guilt, thereby reducing subsequent real-world pro-environmental behavior.

(2) The “environmentalist” labeling reinforces the positive spillover effect mediated by environmental self-identity and attenuates the negative spillover effect mediated by guilt, ultimately shifting the total spillover effect of pro-environmental behavior in the Metaverse on real-world pro-environmental behavior toward a positive spillover.

## Data Availability

The datasets presented in this study can be found in online repositories. The names of the repository/repositories and accession number(s) can be found in the article/supplementary material.
